# HORMAD1 promotes docetaxel resistance in triple negative breast cancer by enhancing DNA damage tolerance

**DOI:** 10.3892/or.2021.8146

**Published:** 2021-07-19

**Authors:** Beige Zong, Lu Sun, Yang Peng, Yihua Wang, Yu Yu, Jinwei Lei, Yingzi Zhang, Shipeng Guo, Kang Li, Shengchun Liu

Oncol Rep 46: Article no. 138, 2021; DOI: 10.3892/or.2021.8089

Following the publication of this article, the authors realized that the published version of Fig. 4A contained an erroneous label; essentially, the information purported to relate to experiments having been performed with docetaxel should not have been included in this figure.

The correctly labelled version of Fig. 4 is shown with the remainder of Fig. 4 on the next page. This change does not affect the data shown in the paper, and the text in the published article did accurately describe the information shown in this figure. The authors sincerely apologize for the error that was introduced during the preparation of this figure, and thank the Editor for allowing them the opportunity to publish a Corrigendum. Furthermore, they regret any inconvenience caused.

## Figures and Tables

**Figure 4.**
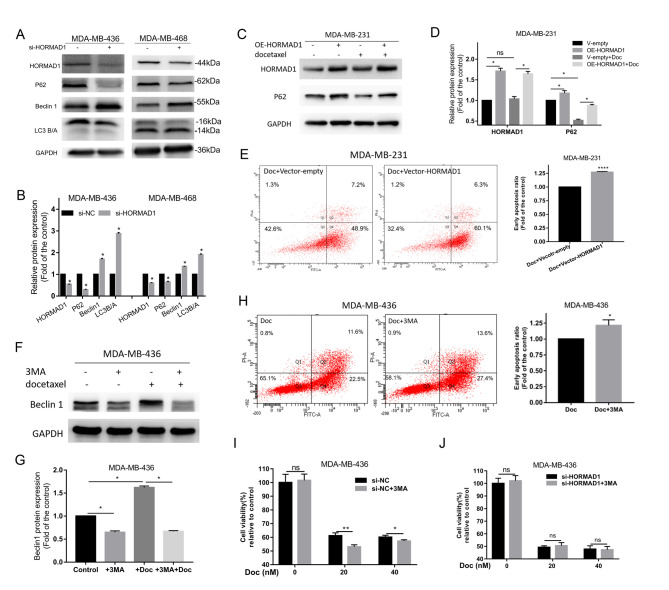
HORMAD1 promotes Doc resistance by not enhancing autophagy. (A and B) Western blot analysis of autophagy markers in MDA-MB-436 and MDA-MB-468 cells. P62 and LC3A were decreased and Beclin 1 was increased in the HORMAD1-knockdown group. (C and D) P62 expression was significantly increased in response to HORMAD1 overexpression in MDA-MB-231 cells. (E) Doc-induced apoptosis for 24 h in MDA-MB-231 cells overexpressing HORMAD1 was higher than that in the control group. (F and G) The addition of 3MA significantly inhibited the expression of Beclin 1, while the addition of Doc significantly increased the expression of Beclin 1. (H) Apoptotic rates induced by Doc for 24 h in MDA-MB-436 cells treated with 3MA were higher than those in the control group. (I) The viability of MDA-MB-436 cells treated with 3MA was lower than that of cells in the control group following exposure to Doc for 24 h. (J) Cell viability of MDA-MB-436 treated with si-HORMAD1 was not altered by treatment with Doc for 24 h, with or without 3MA. GAPDH was used as an internal reference protein. *P<0.05, **P<0.01 and ****P<0.001. HORMAD1, HORMA domain-containing protein 1; Doc, docetaxel; 3MA, 3-methyl-adenine; si-NC, small interfering RNA-negative control; si-HORMAD1, siRNA-HORMAD1; ns, no significance; oe, overexpression.

